# High-light inhibition of two submerged macrophytes in a shallow water experiment

**DOI:** 10.1093/aobpla/plac009

**Published:** 2022-03-04

**Authors:** Jin-Rui Yuan, Zhong-Xi Bai, Shi-Yun Ye, Hui Liu, Yan-Hong Wang, Feng Li, Yong-Hong Xie, An-Guo Gao, Ai-Ping Wu

**Affiliations:** 1 Ecology Department, College of Resources and Environment, Hunan Provincial Key Laboratory of Rural Ecosystem Health in Dongting Lake Area, Hunan Agricultural University, Changsha 410128, China; 2 School of Forestry and Bio-technology, Zhejiang Agriculture and Forestry University, Hangzhou 311300, China; 3 CAS, Key Laboratory of Agro-ecological Processes in Subtropical Region, Institute of Subtropical Agriculture, Changsha 410128, China; 4 Department of Computer Science, Huaihua University, Huaihua 418000, China

**Keywords:** Eutrophication, high-light inhibition, negative growth, photo-damage, photoinhibition, restoration, submerged species

## Abstract

The negative effects, caused by high light, on algae, terrestrial and marine aquatic plants are well documented; those negative effects on freshwater submerged plants are, however, not well known. We determined the negative effects of two common submerged species, *Myriophyllum spicatum* and *Vallisneria natans*, on their growth and reproduction in a shallow water experiment along an irradiance gradient. Our results highlighted that the plant mass, relative growth rate and shoot height of *V. natans* and *M. spicatum*, and root mass and root length:root mass of *M. spicatum* and leaf mass and shoot height:shoot mass of *V. natans* were significantly negatively affected in shallow water with high-light regime (>50 % of full light). While the ramet number of the two species was stimulated by from 20.0 to 36.4 %, and root length, root:shoot, chlorophyll (a:b), chlorophyll (a + b), leaf carbon, nitrogen and phosphorus contents of the two studied macrophytes were not significantly impacted by light. Our results indicated that the high-light inhibition of plant growth was greater on the shoots than on the roots of the plants, although these effects were significantly different between the two studied submerged species and among the measured traits. Accordingly, we should avoid negative effects caused by high light to improve the performance of submerged species when we conduct submerged aquatic vegetation restoration programmes in eutrophic lakes.

## Teaser

Submerged aquatic vegetation (SAV) restoration is an important and applicable technology to retard the eutrophication process of lakes. Accordingly, planting submerged plants in habitats with shallow water is recommended as submerged species are often limited by low light in these eutrophic water bodies. We found that submerged plants could be inhibited by high light if the water was too shallow. Thus, we should trade-off the water level to avoid high-light inhibition effects on submerged species when we conduct SAV restoration programmes in eutrophic lakes.

## Introduction

Aquatic ecosystems are very important to human societies due to their valuable goods and services (Strayer and Dudgeon 2010; Geist 2011; Hilt *et al.* 2017). However, these ecosystems and even the organisms within them are greatly threatened worldwide because of many environmental and anthropogenic factors (Strayer and Dudgeon 2010; Geist 2011; Liu *et al.* 2020). Accordingly, many aquatic plants are rapidly declining and even disappearing from water bodies, especially due to water eutrophication globally (Geist 2011; Zhang *et al.* 2017; O’Hare *et al.* 2018). This is largely because the growth and reproduction of submerged species are limited by low-light availability in eutrophic lakes (Lacoul and Freedman 2006; Schelske *et al.* 2010; Bornette and Puijalon 2011). Similarly, the failure of submerged aquatic vegetation (SAV) restoration and plantings is mainly attributed to low water transparency in these eutrophic lakes (Chen *et al.* 2009; Liu *et al.* 2016; Brezonik *et al.* 2019). Accordingly, planting submerged macrophytes in habitats with shallow water by lowing water is recommended to ensure enough available light for their growth and convenient planting, especially in some ultra-eutrophic lakes (Chen *et al.* 2009; Li and Wang 2013). However, some submerged macrophytes are negatively affected by photo-damage (especially of photoinhibition) even though the light is relatively low at 100 μmol photons m^−2^ s^−1^ (Su *et al.* 2004; Hussner *et al.* 2010; Zefferman 2014). Thus, photo-damage or high-light inhibition of submerged species in shallow water should be considered when SAV restoration is conducted in eutrophic lakes.

Photoinhibition or photoinhibition-dependent responses are the most evident photo-damage or high-light inhibition effects when plants are suffered high-light stress, though other physiological damages, genetic expression and morphological responses (photo-acclimation) occur simultaneously (Szymańska *et al.* 2017; Vialet-Chabrand *et al.* 2017; Patil *et al.* 2020). Photoinhibition occurs when an organism cannot mitigate photoinactivation due to the failure of photoprotection because the photorepair of photosystem (PS) II lags behind the damage of reaction centre proteins (Hanelt *et al.* 2006). Due to rapid light attenuation with water depth (Schelske *et al.* 2010), most research has concentrated on the impact of low light on the growth and photosynthesis of aquatic plants, and the impacts of high light are very scarce (Aguilera *et al.* 2008; Schubert *et al.* 2015). In addition, an apparent photoinhibition and a decline in photosynthetic capacity are frequently observed in higher irradiance investigations (Su *et al.* 2004; Hussner *et al.* 2010; Petrou *et al.* 2013). In these studies, photoinhibition of organisms across terrestrial plants (Míguez *et al.* 2015), algae (Zhang *et al.* 2019) and marine aquatic plants (Petrou *et al.* 2013; Schubert *et al.* 2015) is frequently investigated, while photoinhibition of freshwater submerged macrophytes is not well documented (Su *et al.* 2004; Hussner *et al.* 2010; Zefferman 2014). This might be because light sources in experimental studies are mostly from artificial light (Zefferman 2014). Artificial light, e.g. ultraviolet radiation (UVR), is often used to determine photoinhibition effects on aquatic plants, as photosynthetically active radiation (PAR) and UVR show similar effects of photoinhibition on all types of aquatic plants (Larkum and Wood 1993). Nevertheless, the mechanisms of photoinhibition between PAR and UVR are significantly different (Hanelt *et al.* 2006). Photoinhibition of PAR is directly correlated with a surplus of photosynthetic pigment absorption of radiation, resulting in a loss of active PS II reaction centres (Osmond 1994). However, photoinhibition of UVR is related to the spectral absorption of DNA and proteins rather than the absorption of photosynthetic pigments, causing direct molecular damage (Hanelt *et al.* 2006). Accordingly, the photoinhibition of submerged macrophytes studied by the use of artificial light (including UVR) rather than PAR cannot well demonstrate the actual photoinhibition of submerged macrophytes in nature (Su *et al.* 2004; Hussner *et al.* 2010; Zefferman 2014). Furthermore, photoinhibition effects on submerged macrophytes are focused on their photosynthetic responses, while photoinhibition effects on the growth and reproduction of submerged macrophytes are greatly neglected (Su *et al.* 2004; Zefferman 2014), possibly due to short experimental periods (Hanelt *et al.* 2006; Hussner *et al.* 2010). Moreover, photoinhibition of submerged macrophytes is not easy to observe in the field, as macrophytes can rapidly recover from photoinhibition through their effective photoprotective mechanisms (Hanelt *et al.* 2006; Hussner *et al.* 2010). To address this research gap, it is a need to account for photo-damage (which may include photoinhibition) or negative effects on the growth and reproduction of submerged macrophytes caused by high-light PAR.

In this study, we selected *Myriophyllum spicatum* and *Vallisneria natans* as our study plants, as Liu *et al.* (2020) noted that they could be used to restore submerged macrophytes in shallow eutrophic lakes in the Yangtze floodplain. We conducted a controlled experiment to determine the morphological, physiological and propagation responses of the two submerged species to a light gradient from May to July in 2018. Accordingly, plant mass, relative growth rate (RGR), shoot height, root length, leaf mass, stem mass, root mass, root:shoot, root length:root mass, shoot height:shoot mass, ramet number, chlorophyll (a:b), chlorophyll (a + b), leaf carbon (C) contents, nitrogen (N) contents and phosphorus (P) contents along the light gradient were measured. We hypothesized that (i) the first 13 indices for both species would be negatively affected by high-light conditions because of photo-damage in both the long and short term; (ii) plant chemical composition (C, N, P contents) will be significantly impacted by light conditions during growth; (iii) aquatic plants would accelerate clonal growth in high-light regimes.

## Materials and Methods

### Pot experiment

This experiment was conducted at the Yunyuan Experiment Station (28.11°N, 113.04°E) of Hunan Agricultural University, Changsha, Hunan Province, China, where the temperature ranged from 19 to 37 °C during the experimental period. Two common submerged macrophytes (*V. natans* and *M. spicatum*) were collected from our aquaculture pond. All collected submerged macrophytes were washed and brushed softly with enough distilled water, and then, robust apical unbranched shoots (15 cm in length and similar in morphology; clonal ramet with three leaves for *V. natans*) were cut for planting. Before planting, six individuals of each species were weighed to obtain their initial fresh mass for each individual (*W*_0_). In every pot, four individuals of the same plant species were planted 5 cm deep. Every pot had a circular area of 490 cm^2^ (soil surface), a 15-cm depth and preweighted 5.0 kg of sediment from a local pond (organic matter: 9.2–10.3 g kg^−1^; total N: 0.68–0.82 g kg^−1^, total P: 0.16–0.22 g kg^−1^ and pH: 6.85–7.12, ~18-cm-thick soil layer). Every pot was put in a plastic cylinder with a height of 1.0 m and a volume of 1000 L of water from a nearby pond, and a total of six pots were evenly put in every plastic cylinder. The pH, total N concentration, NO_3_-N concentration, NH_4_-N concentration, total P concentration and PO_4_^3^-P concentration of the water were 7.2, 0.414 mg L^−1^, 0.075 mg L^−1^, 0.037 mg L^−1^, 0.028 mg L^−1^ and 0.009 mg L^−1^ at 25 °C, respectively. Both the total N and P concentrations in water indicated that the cultural water was eutrophic based on the lake trophic status by chemical analyses, especially of total N and P concentrations (Carlson 1977). Additionally, the sediment was collected from a local eutrophic pond and the N and P were very rich for the growth and development of macrophytes. Furthermore, the two studied submerged species take up nutrients mainly from the sediments although they can also gain N and P from the water (Carignan and Kalff 1980; Carignan 1982). Accordingly, we thought that the two macophytes were not nutrient-limited in our experiment. The pots were randomly treated with one of five levels of light using or not using black polyethylene shade cloth to maintain 100 (CK), 75, 50, 30 and 15 % of the full light. The actual amount of light of the 75, 50, 30 and 15 % light treatments, measured with a digital illuminometer ST-101 (SINTEK, China) in full sun, reached their incident PAR at an average of 75, 53, 30 and 17 % of the full light, respectively. Moreover, the PAR at the water surface in different weather conditions was shown in **Supporting Information—Table S1**. Each species for each light treatment was replicated six times (pots). In this experiment, we used two species, five light treatments and six replicates, resulting in a total of 60 pots (10 plastic cylinders).

Plants were harvested 10 weeks later when roots were separated from soil by soaking the pot in water for 40 min and softly washing the soil away. The fresh mass (*W*_1_) of each individual was weighed after rinsing off all sediment and removing the excess water. Then, every plant was separated into roots, stems (only for *M. spicatum* as the stem of *V. natans* is not easily separated) and leaves after its shoot height and root length were determined. Then, the root mass, stem mass and leaf mass of the plant were determined. The total mass of the plant (*W*_1_) was the sum of the root, stem and leaf mass. The root:shoot ratio of a plant was calculated as the root mass divided by the weight of the stem and leaf mass. The RGR of the species was calculated using the formula RGR = (ln*W*_1_ − ln*W*_0_)/*t*, where *W*_0_ and *W*_1_ are the initial mass and final mass of the plants in this experiment, respectively, and *t* is the experimental period (days). The mean value of the four individuals within a pot was considered the value of the pot. Then, the root, stem and leaf were dried at 105 °C for 30 min, oven-dried at 70 °C for 72 h and weighed.

Before drying, the pigments of 0.2 g fresh leaf for each pot were extracted by 80 % aqueous alcohol after grinding. Contents of chlorophyll a and chlorophyll b were measured at 645 and 663 nm after filtration, respectively (Lichtenthaler and Wellburn 1983). Then, all four individuals within a pot were mixed together, and ground to a fine powder with a mortar and pestle. The leaf N and P contents of each powered sample were analysed using the colorimetric method with a TU-1901 spectrophotometer (Beijing Purkinje General Instrument Co., Ltd, China) after being digested in H_2_SO_4_ and H_2_O_2_. The leaf C content of the plant was measured by the dichromate oxidation method of Walkey and Black (Nelson and Sommers 1982).

### Data analysis

To assess the effects of light and species identity on the 15 measured characteristics (excluding stem mass due to no data for *V. natans*), two-way analysis of variance (ANOVA) was used, and the Tukey’s Honestly Significant Difference test (Tukey’s HSD) was used when the raw data were log-transformed. The 15 measured characteristics were categorized as dependent variables, and species and light were categorized as fixed factors when the two-way ANOVA was conducted. Similarly, the differences among the effects of the light treatment on the 16 or 15 measured indices for the two studied macrophytes were determined by one-way ANOVA, and *post hoc* Tukey’s test for light treatments was conducted at the same time. The ANOVA was analysed at the 95 % confidence level, and homogeneity of variances was tested by Levene’s test. Based on the sum of squares (SS) of the two-way ANOVA, variance partitioning can imply the contribution of each source to the variance in the studied indices (Güsewell and Koerselman 2002). The total SS of the ANOVA was equal to the sum of the SS of each factor: SS_total_ = SS_species_ + SS_light_ + SS_species × light_ + SS_error_. Then, the variance contribution of each source was expressed as a percentage of the total SS. All statistical analyses were conducted using the software package R 3.5.2 (R Core Team 2018).

## Results

### Light effects on *V. natans*

**Figure 1.**
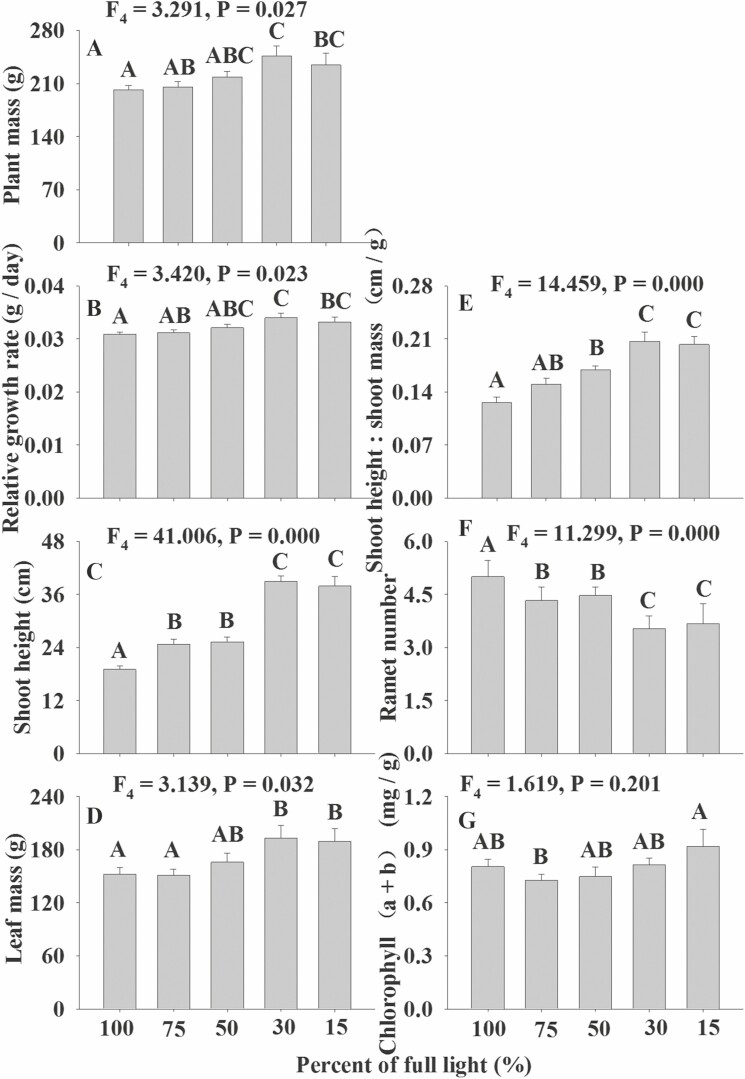
The photo-damage effects on the growth and reproduction of *V. natans* along the light gradient (only the significant traits are shown).

Our results showed no differences among the effects of the five light treatments on the root length, root weight, root:shoot, chlorophyll (a:b), chlorophyll (a + b), root length:root mass, C contents, N contents and P contents of *V. natans* (Fig. 1, *P* > 0.05). However, the effects on plant mass, RGR, shoot height, leaf mass, shoot height:shoot mass and ramet number of *V. natans* showed significant differences among the five light treatments, although the patterns of these six measured indices were not consistent (Fig. 1, *P* < 0.05). Namely, the first five indices initially increased with the reduction in light and then decreased, although the decreases between the 30 and 15 % treatments were not significant (Fig. 1). The ramet number initially decreased with the reduction in light and then increased at lower light levels, although the ramet number between 30 and 15 % irradiance was not significantly different (Fig. 1F).

### Light effects on *M. spicatum*

**Figure 2.**
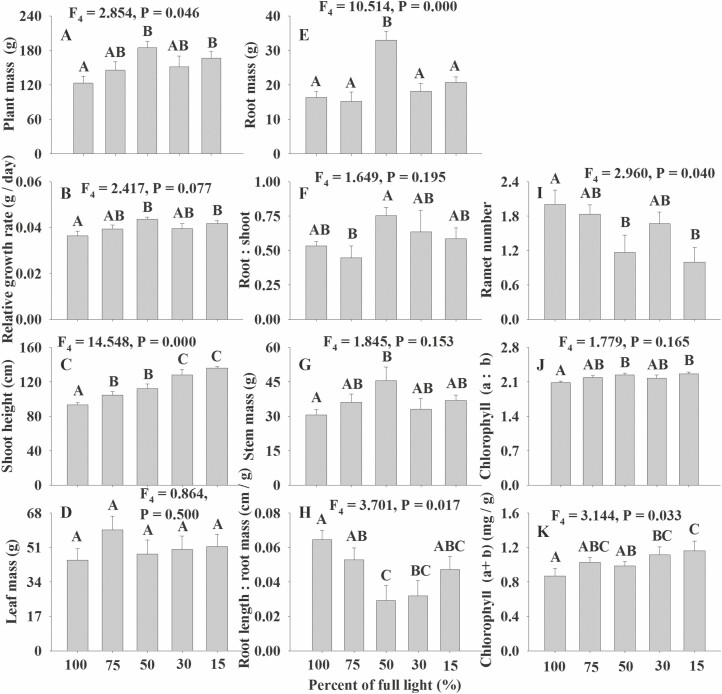
The photo-damage effects on the growth and reproduction of *M. spicatum* along the light gradient (only the significant traits are shown).

Our results indicated no differences among the effects of the five light treatments on the root length, leaf mass, stem mass, RGR, root:shoot, chlorophyll (a:b), shoot height:shoot mass, C contents, N contents and P contents of *M. spicatum* (Fig. 2, *P* > 0.05). However, the effects on plant mass, root mass, shoot height, root length:root mass, chlorophyll (a + b) and ramet number of *M. spicatum* showed significant differences among the five light treatments, although the trends of the six measured characteristics were not identical (Fig. 2, *P* < 0.05). Namely, the plant mass and root mass initially increased with the reduction in light and then decreased at low-light levels while shoot height, and chlorophyll (a + b) increased with the reduction in light (Fig. 2). Similar to that of *V. natans*, the ramet number of *M. spicatum* also decreased across the light gradient (Fig. 2I). The maximum values of plant mass and root mass for *M. spicatum* occurred in the 50 % light treatment and those of plant mass, RGR, shoot height and leaf mass for *V. natans* occurred in the 30 % light treatment (Figs 1 and 2).

### Two-way ANOVA

The differences in plant mass, leaf mass and RGR were significantly different between the two species and among the five different light treatments (Table 1, *P* < 0.05), and no significant interactions between species and light treatment were observed (Table 1, *P* > 0.05), with species and light treatment explaining >58 % of the total variance. The differences in chlorophyll (a:b), shoot height and ramet number were significantly different between the two studied species and among the five different light treatments (except chlorophyll (a:b), Table 1, *P* < 0.05), and significant interactions between species and light treatment were observed (Table 1, *P* < 0.05), with species and light treatment explaining >96 % of the total variance. Root mass showed no significant difference between the two studied species (Table 1, *P* > 0.05), while significant difference between light treatments, and interactions between species and light treatment occurred (Table 1, *P* < 0.05), with light treatment and interactions between species and light treatment explaining >40 % of the total variance. In terms of root length, root:shoot, root length:root mass, shoot height:shoot mass, chlorophyll (a + b), C contents, N contents and P contents, differences were only significantly different between the two studied species (Table 1, *P* < 0.05), and no significant difference among the five different light treatments and interactions between species and light treatment were observed (Table 1, *P* > 0.05), with species explaining >60 % of the total variance except for the indices of shoot height:shoot mass (37.25%), C content (27.98%) and N content (7.33%).

**Table 1.** ANOVA table and percentage (%) of explained variance based on two-way ANOVA for the 15 indices of the two study submerged species in response to the light gradient.

Factor	Percentage (%)	*F*	*P*	Factor	Percentage (%)	*F*	*P*
Plant mass				RGR			
Species (S)	49.25	70.94	**0.004**	Species (S)	54.48	83.26	**0.000**
Light (L)	9.21	3.32	**0.017**	Light (L)	7.21	2.76	**0.038**
S × L	6.83	2.46	0.057	S × L	5.62	2.16	0.088
Error	34.71			Error	32.69		
Shoot height				Root length			
Species (S)	90.00	1732.73	**0.000**	Species (S)	87.35	414.89	**0.000**
Light (L)	6.59	31.72	**0.000**	Light (L)	0.63	0.75	0.565
S × L	0.82	3.94	**0.007**	S × L	1.49	1.77	0.149
Error	2.60			Error	10.53		
Ramet number				Root mass			
Species (S)	95.40	403.92	**0.000**	Species (S)	0.70	0.59	0.446
Light (L)	1.55	8.83	**0.000**	Light (L)	25.60	5.40	**0.001**
S × L	1.41	2.75	**0.038**	S × L	14.42	3.04	**0.025**
Error	1.64			Error	59.28		
Shoot mass				Root:shoot			
Species (S)	85.10	402.02	**0.000**	Species (S)	63.73	106.81	**0.000**
Light (L)	2.60	3.07	**0.025**	Light (L)	3.75	1.57	0.197
S × L	1.72	2.04	0.104	S × L	2.71	1.14	0.351
Error	10.58			Error	29.81		
Chlorophyll (a + b)				Carbon content			
Species (S)	80.41	106.81	**0.000**	Species (S)	27.98	218.22	**0.000**
Light (L)	0.30	1.57	0.197	Light (L)	5.33	0.202	0.936
S × L	0.86	1.14	0.351	S × L	5.76	0.583	0.676
Error	18.43			Error	60.93		
Nitrogen content				Phosphorus content			
Species (S)	7.33	4.38	**0.042**	Species (S)	80.41	22.96	**0.000**
Light (L)	5.14	0.77	0.552	Light (L)	0.30	1.09	0.370
S × L	3.81	0.57	0.686	S × L	0.86	1.18	0.331
Error	83.73			Error	18.43		
Chlorophyll (a:b)				Root length:root mass			
Species (S)	95.83	128.00	**0.000**	Species (S)	80.05	215.91	**0.000**
Light (L)	0.39	0.55	0.697	Light (L)	0.85	0.60	0.664
S × L	3.01	3.97	**0.007**	S × L	0.61	0.42	0.797
Error	0.77			Error	18.49		
Shoot length:shoot mass							
Species (S)	37.25	36.28	**0.000**				
Light (L)	4.64	1.15	0.346				
S × L	6.65	1.63	0.183				
Error	51.47						

Boldfaced values are significant at the *P* = 0.05 level. For all response variables, DF = 4 for light treatment level and light treatment × species, DF = 50 for error and DF = 1 for species.

## Discussion

The measured characteristics of the two studied plants showed different responses along an increasing light gradient. These can be described as negative (possibly due to photo-damage including photoinhibition or to shade adaptation), neutral (no significant differences across the gradient) and positive effects where the measured indices were highest in the highest light. Accordingly, we discuss these three response patterns separately below.

### Negative responses in high light

Consistent with our first hypothesis, our results highlighted that both submerged macrophytes were negatively affected at high light for some measured indices, which were different between the species. For both species, the plant mass, RGR and shoot height were reduced in high-light conditions (Figs 1 and 2) consistent with photo-damage (including photoinhibition) or high-light inhibition. Similarly, Zefferman (2014) found that the biomass (equal to plant mass in our study), RGR and length:biomass ratio of *Elodea nuttallii* were also photoinhibited at full light. These results indicate that the plant mass, RGR and shoot height of submerged plants might be relatively more sensitive to photo-damage than the other measured parameters. The root mass of *M. spicatum* rather than that of *V. natans* was inhibited by high light, which could be because of their different lifeforms. As a rosette-forming species, the effects of currents and waves on *V. natans* (Owens *et al.* 2008) are not as strong as those on a canopy-forming species, *M. spicatum* (Strand and Weisner 2001; Lu *et al.* 2013). Accordingly, *M. spicatum* must invest more resources to its root to anchor the plant avoiding uprooting or serious damage due to its larger canopy (Strand and Weisner 2001; Zhu *et al.* 2012; Lu *et al.* 2013). Thus, we can presume that the root mass of *M. spicatum* is indirectly affected by high-light inhibition based on our experiment. In contrast to the root mass, the leaf mass of *V. natans* rather than that of *M. spicatum* was affected by high-light inhibition (Figs 1 and 2). This might be because that leaf mass constitutes only a very small percentage of the whole shoot mass of *M. spicatum*, whose negative responses of photo-damage (especially of photoinhibition) are mainly through branching and shoot elongating (Zefferman 2014). In contrast, the leaf mass constitutes a great part of the whole shoot mass of *V. natans*, of which the leaf growth is greatly photo-damaged, as observed in our study (Fig. 1D). Additionally, based on the measured indices impacted by high light, it is very likely that the photo-damage effects of submerged macrophytes are mainly on the shoot of the species. It is very possible that photosynthesis occurs almost entirely in the shoot of the plant. However, we do not think that the lower values of chlorophyll (a:b) and chlorophyll (a + b) of *M. spicatum* under high light were caused by photo-damage (Fig. 2). This scenario possibly occurred because a plant should not invest more energy to chlorophyll to improve the effectiveness of its photosynthesis when the ambient light is sufficient, but it must increase its concentration of chlorophyll to enhance its photosynthesis efficiency when the ambient light is limited (Lu *et al.* 2013; Chen *et al.* 2016; He *et al.* 2019), as observed in our experiment (Fig. 2). Nevertheless, the contents of chlorophyll (a:b) and chlorophyll (a + b) of *V. natans* were not significantly affected by the light gradient, which was likely possible because the light compensation point of *V. natans* is much lower than that of *M. spicatum* (Lu *et al.* 2013; Chen *et al.* 2016; He *et al.* 2019). Furthermore, the maximum values of plant mass, RGR, stem mass and root mass of *M. spicatum* at the percentage of light treatment (50%) were higher than those of plant mass, RGR, shoot height and leaf mass of *V. natans* at the percentage of light treatment (30%), which also suggests that the light compensation point of *V. natans* is much lower than that of *M. spicatum* (Lu *et al.* 2013; Chen *et al.* 2016; He *et al.* 2019).

There may be, of course, other mechanisms for the reduced growth of the two studied macophytes in high light (Figs 1 and 2); our results suggest that photoinhibition effects partially result in their reduction of growth as photoinhibition or photoinhibition-dependent responses are the most evident photo-damage effects when plants are suffered high-light stress (Szymańska *et al.* 2017; Vialet-Chabrand *et al.* 2017; Patil *et al.* 2020). Actually, submerged macrophytes are intended to be planted in shallow water by lowing water for enough available light and convenient planting (Chen *et al.* 2009; Li and Wang 2013), although significant wave action occurs in shallow water on shorelines. Thus, these macrophytes may be more likely to suffer the effects of photo-damage (especially of photoinhibition) rather than wave action as observed in our experiment, where no wave action was involved. Accordingly, we should avoid high-light inhibition of aquatic plants when restoration of SAV is conducted, as submerged macrophytes may be impacted by photo-damage (especially of photoinhibition) or high-light inhibition by strong light in shallow water. Thus, we should plant only those species that can grow optimally in shallow water (high light) rather than species that respond negatively to high light.

### Neutral response to the light gradient

Consistent with our second hypothesis, the leaf C, N and P concentrations of the two species were not significantly influenced by the light gradient (Figs 1 and 2; Table 1). Similar results were observed for some submerged macrophytes at different water depths (Li *et al.* 2013) as submerged plants respond similarly to sheltered environments as to deep water depths (Strand and Weisner 2001). This result indicates that the stoichiomestry of submerged species is very conservative (Li *et al.* 2013). As stated above, the contents of chlorophyll (a:b) and chlorophyll (a + b) of *V. natans* showed no significant differences among the light treatments, possibly because the minimum treatment light was far greater than its light compensation point (Morris *et al.* 2004; Lu *et al.* 2013; Chen *et al.* 2016; He *et al.* 2019). Furthermore, root length, root mass and root length:root mass (except for *M. spicatum*) and root:shoot of the studied species were not significantly different over the light gradient, which also suggests that the roots of submerged plants are not as susceptive to light as their shoots, which might be because the roots are underground and cannot directly obtain light.

### Positive responses in high light

In contrast to high-light inhibition, the high-light treatment resulted in increased numbers of ramets in the two species (Figs 1 and 2). However, we do not think that clonal propagation of the studied macrophytes is promoted by high-light availability, as plants can allocate more resources to reproduction when they are exposed to stress (Bonser 2013; Yuan *et al.* 2016). Usually, submerged species can trade-off between their growth and reproduction under adverse conditions (Yuan *et al.* 2016), as observed in our experiment. Our results indicate that the asexual propagation of *V. natans* and *M. spicatum* is stimulated when their growth is inhibited by high light, which is a common strategy for submerged macrophytes to cope with harsh environments (Roff 1992; Bonser 2013; Yuan *et al.* 2016). Accordingly, our results suggest that the two submerged macrophytes trade-off between their growth and reproduction when they are negatively affected by high-light conditions, which is consistent with our third hypothesis.

Generally, our experiment indicated that the two studied species responded differently to light, and even the different determined characteristics of the same species showed different responses to light (Figs 1 and 2; Table 1). The results are in good agreement with the results of many former experiments due to the differences between species and the index specificity within a species (Rae *et al.* 2001; Hanelt *et al.* 2006; Wu *et al.* 2018, 2020; Qi *et al.* 2021). Our results highlighted that the plant mass, RGR, shoot height, ramet number (result because of trade-off between growth and reproduction) and leaf mass were significantly affected by high light although plant species explained most (>49 %) of the total variance in the above five measured indices (Table 1). Furthermore, the effects of high light on plant growth were mainly on the shoots of the plants, as stated above (Table 1). Moreover, we demonstrated that the effects of high light on growth and reproduction of submerged macrophytes could occur in shallow water even though the experimental period is relative long, as observed by Zefferman (2014).

In conclusion, our results highlighted that the plant mass, RGR and shoot height of *V. natans* and *M. spicatum*; root mass of *M. spicatum*; and leaf mass and shoot height:shoot mass of *V. natans* were significantly negatively affected by high light in shallow water. While the ramet number of the two species was stimulated, root length, root:shoot, chlorophyll (a:b), chlorophyll (a + b), leaf carbon, N and P contents of the two studied macrophytes were not significantly impacted by light. Our results indicated that the high-light inhibition of plant growth was mainly on the shoots rather than on the roots of the plants, although these effects were significantly different between the two studied submerged species and among the measured characteristics. Accordingly, we should avoid high-light inhibition effects to improve the performance of submerged species when we conduct SAV restoration programmes in eutrophic lakes.

## Supporting Information

The following additional information is available in the online version of this article—

**Table S1.** The photosynthetically active radiation (PAR) at the water surface in different weather conditions.

plac009_suppl_Supplementary_Table_S1

## Data Availability

Please contact the author for data requests, and the data will be made available upon your request.

## Notes

**Form & Function. Chief Editor**: Kate McCulloh

## References

[CIT0001] Aguilera J , Figueroa FL, Hader DP, Jimenez C. 2008. Photoinhibition and photosynthetic pigment reorganisation dynamics in light/darkness cycles as photoprotective mechanisms of *Porphyra umbilicalis* against damaging effects of UV radiation. Scientia Marina 72:87–97.

[CIT0002] Bonser SP. 2013. High reproductive efficiency as an adaptive strategy in competitive environments. Functional Ecology 27:876–885.

[CIT0003] Bornette G , Puijalon S. 2011. Response of aquatic plants to abiotic factors: a review. Aquatic Science 73:1–14.

[CIT0004] Brezonik PL , Bouchard RW, Finlay JC, Griffin CG, Olmanson LG, Anderson JP, Arnold WA, Hozalski R. 2019. Color, chlorophyll a, and suspended solids effects on Secchi depth in lakes: implications for trophic state assessment. Ecological Applications 29:e01871. 30739365 10.1002/eap.1871

[CIT0005] Carignan R. 1982. An experiment model to estimate the relative importance of roots in P uptake by aquatic macrophytes. The Canadian Journal of Fisheries and Aquatic Sciences 39:243–247.

[CIT0006] Carignan R , Kalff J. 1980. Phosphorus sources for aquatic weeds: water or sediments? Science 207:987–989. 17830461 10.1126/science.207.4434.987

[CIT0007] Carlson R. 1977. A trophic state index for lakes. Limnology and Oceanography 22:361–369.

[CIT0008] Chen J , Cao T, Zhang X, Xi Y, Ni L, Jeppesen E. 2016. Differential photosynthetic and morphological adaptations to low light affect depth distribution of two submersed macrophytes in lakes. Scientific Reports 6:34028. 27694880 10.1038/srep34028 PMC5046178

[CIT0009] Chen KN , Bao CH, Zhou WP. 2009. Ecological restoration in eutrophic Lake Wuli: a large enclosure experiment. Ecological Engineering 35:1646–1655.

[CIT0010] Geist J. 2011. Integrative freshwater ecology and biodiversity conservation. Ecological Indicators 11:1507–1516.

[CIT0011] Güsewell S , Koerselman W. 2002. Variation in nitrogen and phosphorus concentrations of wetland plants. Perspectives in Plant Ecology, Evolution and Systematics 5:37–61.

[CIT0012] Hanelt D , Hawes I, Rae R. 2006. Reduction of UV-B radiation causes an enhancement of photoinhibition in high light stressed aquatic plants from New Zealand lakes. Journal of Photochemistry and Photobiology B: Biology 84:89–102. 10.1016/j.jphotobiol.2006.01.013 16540338

[CIT0013] He L , Zhu T, Wu Y, Li W, Zhang H, Zhang X, Hilt S. 2019. Littoral slope, water depth and alternative response strategies to light attenuation shape the distribution of submerged macrophytes in a mesotrophic lake. Frontiers in Plant Science 10:169. 30842784 10.3389/fpls.2019.00169 PMC6391712

[CIT0014] Hilt S , Brothers S, Jeppesen E, Veraart AJ, Kosten S. 2017. Translating regime shifts in shallow lakes into changes in ecosystem functions and services. BioScience 67:928–936.

[CIT0015] Hussner A , Hoelken HP, Jahns P. 2010. Low light acclimated submerged freshwater plants show a pronounced sensitivity to increasing irradiances. Aquatic Botany 93:17–24.

[CIT0016] Lacoul P , Freedman B. 2006. Environmental influences on aquatic plants in freshwater ecosystems. Environmental Reviews 14:89–136.

[CIT0017] Larkum AWD , Wood WF. 1993. The effect of UV-B radiation on photosynthesis and respiration of phytoplankton, benthic macroalgae and seagrasses. Photosynthesis Research 36:17–23. 24318794 10.1007/BF00018071

[CIT0018] Li Q , Wang GX. 2013. The research on growth and restoration of submerged plants. Beijing, China: China Water Conservancy and Hydropower Press, 1–193.

[CIT0019] Li W , Cao T, Ni LY, Zhang XL, Zhu GR, Xie P. 2013. Effects of water depth on carbon, nitrogen and phosphorus stoichiometry of five submersed macrophytes in an in situ experiment. Ecological Engineering 61:358–365.

[CIT0020] Lichtenthaler HK , Wellburn AR. 1983. Determinations of total carotenoids and chlorophylls a and b of leaf extracts in different solvents. Biochemical Society Transactions 11:591–592.

[CIT0021] Liu H , Zhou W, Li XW, Chu QH, Tang N, Shu BZ, Liu GH, Xing W. 2020. How many submerged macrophyte species are needed to improve water clarity and quality in Yangtze floodplain lakes? Science of the Total Environment 724:138267. 10.1016/j.scitotenv.2020.138267 32247982

[CIT0022] Liu X , Zhang Y, Shi K, Lin J, Zhou Y, Qin B. 2016. Determining critical light and hydrologic conditions for macrophyte presence in a large shallow lake: the ratio of euphotic depth to water depth. Ecological Indicators 71:317–326.

[CIT0023] Lu J , Wang Z, Xing W, Liu G. 2013. Effects of substrate and shading on the growth of two submerged macrophytes. Hydrobiologia 700:157–167.

[CIT0024] Míguez F , Fernández-Marín B, Becerril JM, García-Plazaola JI. 2015. Activation of photoprotective winter photoinhibition in plants from different environments: a literature compilation and meta-analysis. Physiologia Plantarum 155:414–423. 25626882 10.1111/ppl.12329

[CIT0025] Morris K , Harrison KA, Bailey PCE, Boon PI. 2004. Domain shifts in the aquatic vegetation of shallow urban lakes: the relative roles of low light and anoxia in the catastrophic loss of the submerged angiosperm *Vallisneria americana*. Marine and Freshwater Research 55:749–758.

[CIT0026] Nelson DW , Sommers LE. 1982. Total carbon, organic carbon and organic matter. In: Page AL, Miller RH, Keeney DR, eds. Methods of soil analysis. Madison, WI: American Society of Agronomy, 539–579.

[CIT0027] O’Hare MT , Aguiar FC, Asaeda T, Bakker ES, Chambers PA, Clayton JS, Wood KA. 2018. Plants in aquatic ecosystems: current trends and future directions. Hydrobiologia 812:1–11.

[CIT0028] Osmond CB. 1994. What is photoinhibition? Some insights from comparisons of shade and sun plants. In: Baker NR, Bowyer JR, eds. Photoinhibition of photosynthesis, from the molecular mechanisms to the field. Oxford: BIOS Scientific Publication, 1–24.

[CIT0029] Owens C , Smart RM, Dick GO. 2008. Effects of water level fluctuation on *Vallisneria americana* Michx growth. Journal of Aquatic Plant Management 46:117–119.

[CIT0030] Patil S , Prakash G, Lali AM. 2020. Reduced chlorophyll antenna mutants of *Chlorella saccharophila* for higher photosynthetic efficiency and biomass productivity under high light intensities. Journal of Applied Phycology 32:1559–1567.

[CIT0031] Petrou K , Jimenez-Denness I, Chartrand K, McCormack C, Rasheed M, Ralph PJ. 2013. Seasonal heterogeneity in the photophysiological response to air exposure in two tropical intertidal seagrass species. Marine Ecology Progress Series 482:93–106.

[CIT0032] Qi L-Y , Zeng H-Y, Bai Z-X, Wang Y-H, Liu L, Zhong W, Ye S-Y, Fu H, Li F, Shao C-L, Wu A-P. 2021. The effects of biodiversity gradient on plant mass and metabolism of individual submerged macrophytes. Ecological Process 10:38.

[CIT0033] R Core Team. 2018. R: a language and environment for statistical computing. Vienna, Austria: R Foundation for Statistical Computing.

[CIT0034] Rae R , Hanelt D, Hawes I. 2001. Sensitivity of freshwater macrophytes to UV radiation: relationship to depth zonation in an oligotrophic New Zealand lake. Marine and Freshwater Research 52:1023–1032.

[CIT0035] Roff DA. 1992. The evolution of life histories: Theory and analysis. New York: Chapman & Hall publication, 179–241.

[CIT0036] Schelske CL , Lowe EF, Kenney WF, Battoe LE, Brenner M, Coveney MF. 2010. How anthropogenic darkening of Lake Apopka induced benthic light limitation and forced the shift from macrophyte to phytoplankton dominance. Limnology and Oceanography 55:1201–1212.

[CIT0037] Schubert N , Colombo-Pallota MF, Enriquez S. 2015. Leaf and canopy scales characterization of the photoprotective response to high-light stress of the seagrass *Thalassia testudinum*. Limnology and Oceanography 60:286–302.

[CIT0038] Strand JA , Weisner SEB. 2001. Morphological plastic responses to water depth and wave exposure in an aquatic plant (*Myriophyllum spicatum*). Journal of Ecology 89:166–175.

[CIT0039] Strayer DL , Dudgeon D. 2010. Freshwater biodiversity conservation: recent progress and future challenges. Journal of the North American Benthological Society 29:344–358.

[CIT0040] Su W , Zhang G, Zhang Y, Xiao H, Xia F. 2004. The photosynthetic characteristics of five submerged aquatic plants. Acta Hydrobiologica Sinica 28:395–400.

[CIT0041] Szymańska R , Ślesak I, Orzechowska A, Kruk J. 2017. Physiological and biochemical responses to high light and temperature stress in plants. Environmental and Experimental Botany 139:165–177.

[CIT0042] Vialet-Chabrand S , Matthews JSA, Simkin AJ, Raines CA, Lawson T. 2017. Importance of fluctuations in light on plant photosynthetic acclimation. Plant Physiology 173:2163–2179. 28184008 10.1104/pp.16.01767 PMC5373038

[CIT0043] Wu AP , He Y, Ye SY, Qi LY, Liu L, Zhong W, Wang YH, Fu H. 2020. Negative effects of a piscicide, rotenone, on the growth and metabolism of three submerged macophytes. Chemosphere 250:126246. 32097811 10.1016/j.chemosphere.2020.126246

[CIT0044] Wu AP , Liu J, He FF, Wang YH, Zhang XJ, Duan XD, Qian ZY. 2018. Negative relationship between diversity and productivity under plant invasion. Ecological Research 33:949–957.

[CIT0045] Yuan G , Fu H, Zhong J, Lou Q, Ni L, Cao T. 2016. Growth and C/N metabolism of three submersed macrophytes in response to water depths. Environmental and Experimental Botany 122:94–99.

[CIT0046] Zefferman E. 2014. Increasing canopy shading reduces growth but not establishment of *Elodea nuttallii* and *Myriophyllum spicatum* in stream channels. Hydrobiologia 734:159–170.

[CIT0047] Zhang Y , Fu F, Hutchins DA, Gao K. 2019. Combined effects of CO_2_ level, light intensity, and nutrient availability on the coccolithophore *Emiliania huxleyi*. Hydrobiologia 842:127–141.

[CIT0048] Zhang YL , Jeppesen E, Liu IXH, Qin BQ, Shi K, Zhou YQ. 2017. Global loss of aquatic vegetation in lakes. Earth-Science Reviews 173:259–265.

[CIT0049] Zhu G , Li W, Zhang M, Ni L, Wang S. 2012. Adaptation of submerged macrophytes to both water depth and flood intensity as revealed by their mechanical resistance. Hydrobiologia 696:77–93.

