# Supplementary material for: High-light inhibition of two submerged macrophytes in a shallow water experiment
Source: AoB Plants. 2022 Mar 4;14(2):plac009. doi: 10.1093/aobpla/plac009 (PMC8994855; doi:10.1093/aobpla/plac009)
Supplement: plac009_suppl_Supplementary_Table_S1 [file plac009_suppl_supplementary_table_s1.docx]

**Table S1** The photosynthetically active radiation (PAR) at the water surface in different weather conditions.

| Time and weather | Percent of full light | Photosynthetic active radiation (μmol m^-2^ s^-1^) |
| --- | --- | --- |
| May 24th, 2018 at 10: 30 am, sunny, cloudless, 30°C | 100 | 1620-1651 |
|  | 75 | 1246-1260 |
|  | 50 | 854-890 |
|  | 30 | 593-478 |
|  | 15 | 287-325 |
| June 20th, 2018 at 10: 30 am, rainy, 25°C | 100 | 69.8-70.2 |
|  | 75 | 53.9-54.1 |
|  | 50 | 37.2-38.3 |
|  | 30 | 21.3-21.4 |
|  | 15 | 11.8-12.5 |
